# The contribution of environmental exposure to the etiology of autism spectrum disorder

**DOI:** 10.1007/s00018-018-2988-4

**Published:** 2018-12-20

**Authors:** Sven Bölte, Sonya Girdler, Peter B. Marschik

**Affiliations:** 1 Department of Women’s and Children’s Health, Karolinska Institutet & Child and Adolescent Psychiatry, Stockholm Health Care Services, Center of Neurodevelopmental Disorders (KIND), Centre for Psychiatry Research, Stockholm County Council, Stockholm, Sweden; 2 Curtin Autism Research Group, School of Occupational Therapy, Social Work and Speech Pathology, Curtin University, Perth, WA Australia; 3 Department of Child and Adolescent Psychiatry and Psychotherapy, University Medical Center Göttingen, Göttingen, Germany; 4 iDN-interdisciplinary Developmental Neuroscience, Department of Phoniatrics, Medical University of Graz, Graz, Austria

**Keywords:** Autism, Neurodevelopmental disorders, Environment, Etiology, Genes, Twins

## Abstract

Autism spectrum disorder (ASD) is a neurodevelopmental condition of heterogeneous etiology. While it is widely recognized that genetic and environmental factors and their interactions contribute to autism phenotypes, their precise causal mechanisms remain poorly understood. This article reviews our current understanding of environmental risk factors of ASD and their presumed adverse physiological mechanisms. It comprehensively maps the significance of parental age, teratogenic compounds, perinatal risks, medication, smoking and alcohol use, nutrition, vaccination, toxic exposures, as well as the role of extreme psychosocial factors. Further, we consider the role of potential protective factors such as folate and fatty acid intake. Evidence indicates an increased offspring vulnerability to ASD through advanced maternal and paternal age, valproate intake, toxic chemical exposure, maternal diabetes, enhanced steroidogenic activity, immune activation, and possibly altered zinc–copper cycles and treatment with selective serotonin reuptake inhibitors. Epidemiological studies demonstrate no evidence for vaccination posing an autism risk. It is concluded that future research needs to consider categorical autism, broader autism phenotypes, as well as autistic traits, and examine more homogenous autism variants by subgroup stratification. Our understanding of autism etiology could be advanced by research aimed at disentangling the causal and non-causal environmental effects, both founding and moderating, and gene–environment interplay using twin studies, longitudinal and experimental designs. The specificity of many environmental risks for ASD remains unknown and control of multiple confounders has been limited. Further understanding of the critical windows of neurodevelopmental vulnerability and investigating the fit of multiple hit and cumulative risk models are likely promising approaches in enhancing the understanding of role of environmental factors in the etiology of ASD.

## Introduction

Autism spectrum disorder (ASD) is an early onset neurodevelopmental condition defined in the DSM-5 by alterations in social communication and interaction in conjunction with repetitive, inflexible behaviors and circumscribed interests causing significant impairment in major life areas [1, 2] and reduced quality of life [3]. Neurodevelopmental conditions provide an umbrella term inclusive of disorders arising from extreme variations (neurodiversity) or qualitative alterations in the maturation, architecture, and functioning of the developing brain and are present in a substantial minority (10–15%) of the general population [4]. The DSM-5 criteria for ASD includes a specifier recommending that the potential role of medical and genetic conditions, and environmental factors associated with atypical neurodevelopment leading to ASD be considered. Neurodevelopmental changes in ASD impact broadly on cognitive abilities (e.g., executive function, top-down processing, social cognition), the social brain and other neural structures [5–8]. ASD also affects other major physiological systems including the immune, endocrine, and gut microbiota systems [9–12]. The cumulative impact of ASD on health related outcomes is evidenced by an increased risk for somatic and psychiatric illness, and premature mortality [13–15]. Prevalence estimates and diagnoses rates of ASD have risen substantially in the last two decades reaching 1–2.5% [16, 17], with some regions reporting even higher figures [18]. While diagnosis in males exceeds that of females threefold, the rate of ASD among girls and women is likely underestimated by male-centric operationalization of the autism phenotype, female “camouflaging” and internalizing psychiatric comorbidity [19, 20]. Increased understanding of the autism phenotype has underpinned the development of effective evidence-based behavioral interventions [21], and poor etiological insight limits the development of biological treatments or the discovery of a “cure” [22]. The conceptualization of ASD as a psychiatric disease has been challenged by evolving societal perceptions and increasing tolerance of neurodiversity, and recognition of the role of environmental factors in supporting the functioning [23].

## Nature and nurture

Although there is wide recognition that ASD has multiple causes, both genetic and environmental in origin, precise understanding of the exact mechanisms underpinning atypical neurodevelopment is lacking. Autistic traits and (subclinical) broader phenotypes of ASD are heritable and continuously distributed in the general population, with etiologies overlapping with clinical phenotypes [24]. Genome sequencing data indicates there are hundreds of genes associated with ASD, both common and rare (inherited and de novo), with many shared with other neurodevelopmental, psychiatric, and neurological conditions [25]. Though the clinical utility of genetic evidence is presently limited, it is evolving, enabling in some cases genetic explanations of ASD, estimation of the likelihood of familial recurrence, and identification of other associated genetic risks [26]. While heritability estimates for ASD range from 38 to 55% and upwards to 95% [27, 28], recent twin and family studies suggest heritability plays a smaller role than previously thought, indicating a greater role for environmental factors [29, 30]. While one twin study found shared environment plays a major role in ASD etiology [30], the majority of family and twin studies suggest non-shared environmental factors, or factors unshared between family members that make them dissimilar, are more influential. However, identifying specific non-shared environmental factors is challenging given they extend beyond aspects of nurturing, to factors including measurement error, social chance, random biological noise, immune reaction and neuroinflammation, and epigenetic and genetic differences in identical twins [31]. Evidence of non-shared environmental influences has been found across the life span and autism spectrum, from autistic traits to extreme clinical phenotypes of ASD. Complicating the deciphering of the influence of non-shared environmental factors in the etiology of ASD is the fact that their key mechanism is likely cumulative frequency, rather than single causal agents [32]. Given monozygotic twins share 100% of their genetic variation at a DNA sequence level and dizygotic twins share on average 50%, twin studies provide a unique opportunity for modeling the relative contribution of environmental factors and genetics to ASD phenotypes. Comparing monozygotic and dizygotic twin pairs and their phenotypic concordance and discordance enables investigation of the genetic and environmental contributions (both shared and non-shared) to presentations of ASD (ACE model) [33].

The environment can be both causal if it is harmful and precedes ASD, mediating if it influences the causal chain between a genetic predisposition and ASD, moderating if it impacts the severity of autism, and protective if it decreases the risk of ASD. The biological environment comprises all chemical, bacterial, viral, or physical environmental influences and exposures, directly and primarily acting on the physiology of the individual. Psychosocial environmental factors denote the psychological, social, and cultural environments that primarily act on mental functions and secondarily on physiology. Understanding of the causal role of environmental factors in the etiology of ASD can potentially inform both primary prevention and evidence-based interventions. While the environment is clearly key in mediating avoidable negative outcomes and of paramount significance in secondary and tertiary interventions and supporting autistic individuals in everyday life, the present article centers on its role in ASD etiology or putative ASD causality. Although there it is no doubt as to the role of the psychosocial environment in moderating ASD, its casual role in rare cases of early, extreme persistent deprivation and hospitalization on psychopathology including autistic-like patterns cannot be dismissed. Finally, while research has examined the role of environmental factors in increasing autism risk, emerging research balances this focus, reconsidering the environment as a potentially protective factor in the etiology of ASD.

Research examining the genetic and environmental contributions to the etiology of ASD has largely examined factors in isolation, rather than considering the role of gene–environment interactions through processes such as epigenetic dysregulation. Epigenetic mechanisms modify gene expressions controlled by factors other than DNA sequencing and are potentially reversible. There is evidence that epigenetic mechanisms [34, 35], such as DNA methylation, play a significant role in ASD etiology in combining genetic and environmental factors that dysregulate neurodevelopmental processes [36, 37]. A body of emerging evidence points to multiple hit and threshold models, integrating both genetic and environmental contributions such as the three-hit concept of vulnerability and resilience, and the Trigger–Threshold–Target model [38, 39], as fruitful approaches in understanding the etiology and development of the autism phenotype.

## Environmental factors

Investigated biological environmental risk factors in ASD include maternal and paternal age, fetal environment (e.g., sex steroids, maternal infections/immune activation, obesity, diabetes, hypertension, or ultrasound examinations), perinatal and obstetric events (e.g., hypoxia), medication (valproate, selective serotonin reuptake inhibitors), smoking and alcohol use, nutrition (e.g., short inter-pregnancy intervals, e.g., vitamin D, iron, zinc, and copper), vaccination, and toxic exposures (air pollution, heavy metals, pesticides, organic pollutants). Surprisingly, the role of potentially protective factors such as folate and fatty acid intake and levels are far less frequently examined. Considering the psychosocial environment, the relevance of extreme psychosocial institutional deprivation and maternal stress during flight and immigration has been discussed in relation to atypical behavior development, including autistic features. While there are many postulated mechanisms through which these environmental factors might generate autistic behaviors and clinical variants of ASD, inflammation and immune activation, oxidative stress, hypoxia, and endocrine disruptions are likely the most pivotal in contributing to atypical neurodevelopment. Although the relevance of these factors may not be directly causal, but confounded by genetic factors, understanding is limited by the paucity of research examining gene–environment interactions.

This review summarizes our understanding of the role of environmental factors and their postulated mechanisms in the etiology of ASD. Although several reviews in this field have been published in recent years [40–42], the present state-of-the art review extends those previous in updating the literature, capturing studies to August 2018, providing additional methodological points of discussion, and including recent research examining the significance of environmentally mediated elemental metal dysregulation in autism etiology. Of note, while the DSM-5 definition is used today and soon ICD-11 [https://icd.who.int/browse11/l-m/en] will be employed in international clinical practice, many studies reviewed in this article used DSM-IV-TR criteria of ASD and considered specific ASD diagnoses within the DSM-IV-TR definition.

### Parental age

The significance of advanced parental age is a well-established risk factor for chromosomal aberrations, such as advanced maternal age in Down syndrome. There is accumulating evidence of the relevance of older parental age in the etiology of psychiatric and neurodevelopmental conditions [43] including bipolar disorder, schizophrenia, substance use disorders, ADHD, and ASD [44]. While various hypotheses have been posed as to the biological mechanisms of maternal and paternal age effects, an association between advanced parental age and increasing likelihood of malign de novo mutations has been suggested [45]. This is most likely explained by a cumulating risk for mutations during spermatogenesis across the life span [46]. Indeed, de novo mutations associated with ASD are more often paternal than maternal [47], with some evidence of linked autism risk in offspring of older fathers with detected age-related DNA methylation changes in their sperm [48]. Interestingly, these effects may even be intergenerational, with advanced grandparent paternal age on both mother’s and father’s side linked to ASD, suggesting that parental age-related risk might accumulate over generations [49]. Neurobiologically, increased paternal age has been associated with reduced cortical thickness of the right ventral posterior cingulate cortex [50].

It has also been postulated that the increasing risk of ASD with advancing age is explained by males with autism risk, in the form of a subclinical broader autism phenotype, being more likely to father children later in life. If this is the case, the increasing risk of ASD with advancing paternal age could be explained by genetic predisposition, rather than biological aging. However, this hypothesis is yet to be corroborated [51]. Countering this theory is evidence that young parental aged is associated with some neurodevelopmental disorders, for instance ADHD [52], a disorder often comorbid to ASD [53]. Here, psychosocial factors rather than biological, such as an unhealthy lifestyle, and economical and educational disadvantage associated with early parenthood, have been put forward as explanations for these associations [54].

Parental age-related risk in ASD has been found in cohorts across multiple geographic regions, with evidence that parental age-related risks for ASD presents independently for maternal and paternal age. There is evidence that parental age-related risk is at its highest in offspring where both the mother and father are advanced in age, and that there is an increasing risk of ASD for couples with greater age differentials [55]. It is also possible that advanced paternal age generates a higher risk for female offspring and higher maternal age for male offspring [56, 57]. Recently, a meta-analysis of 27 observational studies investigating the association between advanced parental age and risk of autism [58] found that the lowest parental age category was associated with a reduced risk of autism in offspring [odds ratio (OR) 0.89, 95% confidence interval (CI) 0.75–1.06] and OR 0.81 (95% CI 0.73–0.89) for mothers and fathers, respectively. Further, the highest parental age category was associated with an increased risk of autism in the offspring, with ORs 1.41 (95% CI 1.29–1.55) and 1.55 (95% CI 1.39–1.73) for mothers and fathers, respectively. Dose–response meta-analysis methods found no association between maternal age and reduced risk of autism (OR 0.93, 95% CI 0.69–1.24), but a decrease of 10 years in paternal age was associated with a 26% reduced risk of autism (OR 0.74, 95% CI 0.64–0.86). An increase of 10 years in maternal age was associated with an 18% higher risk of autism (OR 1.18, 95% CI 1.10–1.26), and an increase of 10 years in paternal age was associated with a 21% higher risk of autism (OR 1.21, 95% CI 1.18–1.24).

### Fetal environment

Numerous environmental prenatal exposures present within the immediate environment of the developing fetus such as sex hormone alterations, maternal obesity, diabetes, hypertension, infections and immune activity, and ultrasound exposure have been considered in the context of ASD etiology. While the origins of these risks might be in genetic disposition, environmental interactions involving both the mother and fetus with the potential to compromise the fetal–maternal–placental system cannot be ignored. Many of these factors may be the product of the combination of several underlying pathophysiological processes, such as the negative effects of imbalanced fetal sex hormone exposure during critical time windows on gene transcription and expression [59, 60], and subsequent neurotransmitter, neuropeptide, or immune pathways [61]. Obesity bears an independent risk for obstetric complications, coronary heart disease, being overweight, diabetes, and several other medical conditions in the offspring [62]. Maternal obesity is also assumed to impact the brain development and cognitive functions of offspring [63]. Severe maternal obesity and high-fat diet might impact on fetal and offspring neurodevelopment, through processes including low-grade neuroinflammation, increased oxidative stress, insulin resistance, glucose, and leptin signaling, dysregulated serotonergic and dopaminergic signaling, perturbations in synaptic plasticity, and altered DNA methylation patterns [64, 65]. These and additional risks for neurodevelopment are amplified in the presence of co-occurring diabetes [66]. Hypertension during pregnancy contributes substantially to perinatal morbidity and mortality of both the mother and her child [67]. Hypertension can lead to sequelae of adverse utero conditions, potentially altering fetal development and increasing the risk of long-term vascular, cognitive, and psychiatric outcomes in the offspring. High blood pressure is the primary driver of these adverse outcomes. This is particularly problematic when it is associated with preeclampsia, which presents with significant amounts of protein in the urine and risks of red blood cell breakdown, low blood platelet count, impaired liver function, kidney dysfunction, swelling, shortness of breath due to fluid in the lungs, and visual disturbances [68]. Infection during pregnancy activates the maternal immune system, triggering cytokine signaling, passing through the placenta, and possibly causing numerous adverse neural effects in the developing fetal brain [69]. Though not established in human studies, animal studies have linked ultrasound exposure in utero to alterations in neuroanatomy and function, for example in the hippocampus [70].

#### Sex steroids

Regarding hormonal alterations, it has been hypothesized that high fetal exposure to sex steroids may contribute to ASD risk [71]. This is linked to the male brain theory of autism which claims that autism can be characterized as an extreme variant of the male phenotype on the cognitive and other levels [72]. Evidence supporting this notion is apparent in the finding that fetal testosterone influences individual differences in typical development in eye contact behaviors, vocabulary size, restricted interests, mentalizing, empathy, systemizing, attention to detail, and autistic traits [59]. In line with this theory, neuroimaging studies indicate that fetal testosterone affects individual differences in structural and functional brain development. These patterns are consistent with those seen in sexual dimorphism, autism, and other sex-biased developmental conditions [73–75]. A genetic study of autism found evidence that single nucleotide polymorphisms in sex steroid synthesis genes (ESR2, CYP11B1, CYP17A1, CYP19A1) were associated with autism traits and autism without intellectual disability and good verbal skills [76]. A study using a Danish Historic Birth Cohort and Danish Psychiatric Central Register of amniotic fluid samples of males measured concentration levels of sex steroids (progesterone, 17α-hydroxy-progesterone, androstenedione and testosterone) and cortisol using liquid chromatography–tandem mass spectrometry. Principal component analysis showed that a generalized latent steroidogenic factor accounted for the majority of data variance, with the autism group showing elevations across all hormones on the latent factor [77].

Fetal testosterone exposure is one of several hypotheses which attempts to explain the male preponderance of neurodevelopmental disorders, especially in ASD [61]. Polycystic ovary syndrome (POS), a syndrome affecting at least 5% of women of child-bearing age, drives altered prenatal sex hormone exposure leading to a pattern of elevated androgens in females and has been examined in the context of ASD [78]. A nested total population study of Swedish children aged 4–17 years (*n* = 23, 748 ASD, 208,796 controls) showed increased odds for ASD for both female and male offspring (OR 1.59, CI 95% 1.34–1.88) of mothers with POS, with comorbid obesity further increasing the odds for autism (OR 2.13, 95% CI 1.46–3.10) [60]. Another investigation, underpinned by the same sample, reported an increased autism risk in offspring in presence of maternal hirsutism, another condition associated with hyperandrogenism (OR 1.26–1.64; CI 95% 0.94–2.83) [79]. A further study examining autistic traits in offspring of mothers with POS showed higher levels of these traits in daughters, but not sons, compared to unaffected mothers [80]. Finally, in this line, it has been both reported that women with POS themselves have an elevated rate of ASD (OR 1.55, 95% CI 1.32–1.81) [81], and that women with ASD are at risk for disorders related to steroids [82].

#### Obesity

Adiposity is a common global health condition, and while national rates vary greatly about 20% of adults worldwide are severely overweight [83]. Mixed findings have been reported in relation to the association between maternal weight and risk of ASD, with the overall effect of obesity on autism and neurodevelopment remaining unclear [84]. A Swedish study employing matched sibling analysis reported no significant association between maternal obesity and offspring risk of autism [85]. Interestingly, children born to mothers who were both obese and underweight were at higher risk of ASD [86], indicating that extreme weight at both ends of the weight spectrum might be associated with autism. A recent review summarizing the associated risk of weight for autism and other neurodevelopmental disorders across 32 articles and 36 cohorts showed that compared with mothers of normal weight, the offspring of obese and overweight mothers had a 17% increased risk of experiencing any neurodevelopmental disorder (OR 1.17, 95% CI 1.11–1.24) and a 36% increased risk for ASD (OR 1.36; 95% CI 1.08–1.70) [87]. Extending this research, additional studies show excess risk for autism in the presence of maternal obesity when women gain additional weight during pregnancy [88].

#### Diabetes

Studies examining the effect of maternal diabetes on autism in offspring have yielded inconsistent results. A recent systematic literature review and meta-analyses synthesizing 16 studies [89] demonstrated additional risk for autism in the presence of maternal diabetes (relative risk =  1.48, 95% CI 1.26–1.75). While high levels of variation in study outcomes and publication bias were detected, these disappeared when meta-analysis was restricted to case–control studies, with the risk of ASD increasing by 62% among diabetic mothers, compared with non-diabetic mothers. There is evidence that timing might be significant in the association between maternal diabetes and offspring with ASD. A retrospective study of 322,323 singleton Californian children born at 28–44 weeks examined the effect of intrauterine exposure to preexisting type 2 diabetes and gestational diabetes. It reported exposure to maternal gestational diabetes mellitus diagnosed by 26 weeks’ gestation increased the risk of ASD in offspring by 42% [90].

#### Hypertension

At a population prevalence of approximately 10%, high blood pressure disorders are one of the most common pregnancy complications [91]. Theses disorders include chronic hypertension (essential/secondary), white-coat hypertension, masked hypertension, transient gestational hypertension, gestational hypertension, and preeclampsia (de novo or superimposed on chronic hypertension), with pregnancy-related onset typically occurring in the second trimester [92]. A recent systematic review and meta-analysis examining the association between hypertensive disorders of pregnancy and risk of neurodevelopmental disorders in offspring identified 20 studies estimating the risk of ASD, 11 of these with adjusted estimates covering 777,518 participants with a pooled OR of 1.35 for ASD risk (95% CI 1.11–1.64) [93].

#### Infections and immune activation

Since the detection of the association between autism and congenital rubella infection, the role of infections and the immune system in the etiology of autism has been debated [94, 95]. Accumulating evidence suggests that the immune system and abnormal immune function, including inflammation, cytokine dysregulation, and anti-brain autoantibodies, influence trajectories of autism, playing a role in its etiology in at least a subset of cases. In addition to rubella, there are a number of other maternal viral and bacterial infections associated with ASD risk [96, 97]. In particular, maternal influenza bears a twofold risk for autism in offspring [98]. While maternal infection in the presence of fever correlates with risk of ASD, this is attenuated by the use of antipyretic drugs.

A Swedish nationwide register-based birth cohort, born from 1984 to 2007 with follow-up through to 2011 of 2,371,403 persons with 24,414 ASD cases, identified a 30% increase for ASD associated with any maternal inpatient diagnosis of infection [99]. Increased risk for ASD was associated with infection in all trimesters of pregnancy suggesting no effect of timing, contrasting the findings of previous research indicating the timing of infection during pregnancy was relevant [96]. There is also evidence that infections increase the risk for ASD with co-occurring intellectual disability [99]. Although it has long been suggested that cytomegalovirus infection is associated with ASD its contribution to risk remains unclear, with a recent systematic review of this literature and meta-analyses of three observational studies finding that while there was a high rate of the cytomegalovirus in ASD cases, validity was seriously hampered by the low number of events in all studies [100].

The relevance of the pathogenesis of maternal infection to ASD risk may not be associated with the presence of viruses or bacteria per se, but in the immune response they invoke, a conclusion supported by research identifying elevated inflammatory markers and antibodies in pregnant women with autistic offspring [101, 102]. Additional support for the maternal immune activation hypothesis is available from rodent models of neurodevelopmental disorders, with direct infection in dams associated with behavioral changes in offspring, including those relevant to autism such as reduced socialization and vocalizations [103, 104]. Similar observations have been made following maternal immune activation in rhesus macaques [105]. While mounting evidence points to the role of maternal immune activation in ASD risk, refining animal models to enable understanding of the role of timing in prenatal immune challenge, and paired and behavioral phenotyping, would potentially improve the reproducibility of results and maximize the translation of findings to understanding ASD [106].

Although the hypothesis of harmful immune response is well established, what remains less clear is how this response affects the fetus directly. While inflammatory or regulatory cytokine profiles are postulated to have a role in the risk of several neurodevelopmental disorders including autism, through the disruption of cytokine levels, observations to date are limited to rodent models [107, 108]. Several pathways to ASD through cytokines have been suggested: a maternal pathway, whereby cytokines from the mother cross the placenta; a placental pathway, where maternal immune activation leads to inflammation and cytokine production in the placenta; and a fetal pathway, through which maternal immune activation results in immune and gene dysregulation in the fetus itself [109]. The significance of serum or plasma maternal antibodies may not be limited to a single ‘window of infection’, with evidence that these are not transient, but persist for many years beyond the infection [110], raising the possibility that infections or auto-immune conditions prior to conception present a risk for ASD. Consistent with this line of thought, a study of the Simons Simplex Collection reported that mothers of children with ASD were four times as likely to have circulating antibodies [111].

The immune activation paradigm is underpinned by findings from exposure models to maternal autoantibodies. Applying injections of serum containing antibodies into pregnant mice yielded support for their causality in neurodevelopmental adversity, with offspring displaying reduced behavioral exploration, motor control and sociability, higher anxiety, sensory alterations, and stereotypies compared to offspring of control dams [112–115]. Evidence corroborating these findings can be found in maternal antibody models in macaques, with antibody exposure causal in increasing brain growth and total cerebral volume [116, 117], a well-established endophenotype of ASD [118].

#### Ultrasound

While medical ultrasound is generally considered safe, some studies have hypothesized that obstetric diagnostic sonography is detrimental to neurodevelopment and may also pose an ASD risk. Although an older systematic review found no associated risks for obstetric diagnostic sonography, it highlighted that this conclusion was not definitive, as longitudinal studies of neurodevelopmental outcomes were lacking [119]. A study applying diagnostic ultrasound to pregnant mice yielded less prosocial behaviors in offspring compared to sham-exposed controls [120]. Paralleling this research is the finding that ultrasound may have a role in a multiple hit model of autism, in assaying a possible relationship between symptoms of autism, ultrasound exposure during the first trimester of pregnancy and a genetic predisposition to ASD. Consistent with this notion, findings drawn from the Simon’s Simplex Collection report that in male children with ASD, copy number variations and exposure to ultrasound was associated with lower non-verbal IQ and more repetitive behaviors, relative to control children [121].

### Perinatal risk factors

There is a long history of research examining a large number of perinatal factors and their association with autism phenotypes including prematurity, cesarean delivery, low birth weight, low Apgar score, and hypoxia. While many of these factors may have a role in autism risk, they are unlikely to be primarily causal, but rather comprise part of the epiphenomena of genetic autism disposition, with familial autism load itself increasing the likelihood of obstetric complications [122]. Clarity is lacking in regard to the load each of these factors bear in autism, with no specific pregnancy complication consistently connected to ASD and perinatal risk shared with other neurological, psychiatric, and neurodevelopmental disorders. Recently, several reviews and meta-analyses have attempted to synthesize these findings. An early review examined 60 obstetric factors finding that abnormal presentation, umbilical cord complications, fetal distress, birth injury or trauma, multiple birth, maternal hemorrhage, summer birth, low birth weight, small for gestational age, congenital malformation, low 5-min Apgar score, meconium aspiration, neonatal anemia, ABO or Rh incompatibility, and hyperbilirubinemia were associated with ASD risk [123]. A more current review found an increased risk for autism associated with cesarean delivery, gestational age ≤ 36 weeks at birth, induced labor, no labor, breech presentation, and fetal distress, although most odds and relative risk ratios were modest [124]. Parity of four or more children was highlighted as a factor connected to decreased autism risk in one study. Of note, across meta-analyses models complications resulting from hypoxia emerged as the most consistent factors associated with ASD risk.

### Medication

The safety of many medications in pregnancy and lactation is yet to be established, with the majority of therapeutic decisions made during pregnancy underpinned by a paucity of evidence. Often studies examining the effects of medication on offspring are confounded by illnesses, behaviors, and other risk factors associated with psychiatric illness in mothers, including risks linked with untreated psychiatric illness during and after pregnancy. In the ASD literature, antidepressive and anticonvulsive medications have emerged as medications of potential relevance or interest.

#### Valproate

Valproic acid (VPA) or 2-propylpentanoic acid has long been used clinically as a treatment for epilepsy and as a mood stabilizer in bipolar disorder. The use of valproic acid in pregnancy poses multiple risks for offspring including congenital malformations, developmental delay, and cognitive malfunction [125]. Animal models demonstrate that exposure to valproate impacts both short- and long-term neurodevelopmental trajectories, interfering with neural migration pathways at critical points during embryonic development, and potentially contributing to neural tube defects [126, 127]. In humans epigenetic mechanisms implicated in ASD may be a key mechanism through which valproate influences neurodevelopment [34–37]. Recently, a large comparative systematic review and meta-analysis of 29 cohort studies including 5100 infants examined the impact of using antiepileptic drugs during pregnancy or breast feeding on the neurodevelopment of infants, reporting that only valproate was associated with more children experiencing cognitive developmental delay compared with controls (OR 7.40, 95% CI 3.00–18.46). In a subset of studies examining autism risk (5 cohort studies, 2551 children, 12 treatments), this risk was amplified (OR 17.29, 95% CI 2.40–217.60) [128].

#### Selective serotonin uptake inhibitors (SSRIs)

Depression is one of the most commonly occurring mental disorders worldwide, with 10% of women experiencing depression during pregnancy, and a subgroup of up to 10% of these of women in European countries receiving SSRI treatment during gestation [129]. SSRIs cross the placenta barrier, potentially triggering a cascade of adverse effects including reduced serotonin uptake, reduced uterine blood flow, and hypoxia resulting in brain damage. A systematic review of the literature aiming to assess the association between ASD and fetal exposure to antidepressants during pregnancy, from preconception and across each trimester of pregnancy, included ten studies with six case–control studies (117,737 patients) in a meta-analysis [130]. Findings revealed a positive association between SSRI exposure and ASD, consistent across all trimesters (OR 1.81; 95% CI 1.49–2.20), which while partially mitigated by controlling for past maternal mental illness (OR 1.52; 95% CI 1.09–2.12) remained significant. In line with these findings, a Swedish epidemiological study published subsequently to the aforementioned review reported that the risk posed by SSRI exposure may not be solely a byproduct of confounding variables. However, and importantly, the authors stressed that the absolute risk of autism linked with SSRI use was small, and that at a population level abstaining from SSRIs during pregnancy would probably prevent few cases of autism [131]. The reported association between SSRI use and ASD etiology has also been recently challenged by a Canadian retrospective cohort study drawing from 35,906 singleton births finding no association between SSRI exposure in utero and ASD [132]. Research has not found evidence that *paternal* SSRI use around conception increases autism risk [131, 133, 134].

### Smoking and alcohol

It has long been recognized that maternal (and paternal) lifestyle and substance use patterns impact fetal and infant development, with smoking and alcohol consumption among the most extensively researched and widespread [135, 136]. In a multitude of countries, the rates of smoking and alcohol use are decreasing [137]. Smoking exposes a developing fetus to many risks including thousands of potentially harmful chemicals and oxygen deprivation, collectively causing changes in neurotransmitter activity within the developing brain [138, 139]. Ethanol consumption during pregnancy can trigger multiple forms of neurodevelopmental damage, including fetal alcohol syndrome in cases of heavy drinking [130, 140, 141].

Research has consistently shown that both smoking and alcohol use in pregnancy are associated with neurological, psychiatric and neurodevelopmental disorders, including those often comorbid to ASD, such as ADHD [142]. However, specifically for autism phenotypes, the evidence is inconsistent and overall rather weak. Several studies have reported an association between smoking and increased risk for ASD with intellectual disability, but not without [143, 144]. Two meta-analyses undertaken in in 2015, both inclusive of 15 studies, showed no evidence for smoking as a risk factor in ASD, even after correcting for multiple confounds including socioeconomic status and parental psychiatric history [145, 146]. However, these findings must be interpreted with caution given that the majority of the primary research summarized in these meta-analyses failed to be adjusted for relevant confounders such as birth weight and employed self-report data collection methods likely biased by social desirability [147].

A more recent meta-analysis employing population-based smoking metrics as moderators pointed to the importance of investigating paternal and secondhand smoking exposure, in addition to maternal smoking, in understanding the risk smoking bears in ASD [148]. Research examining the risk that maternal alcohol consumption poses to autistic behaviors has largely focused on the context of fetal alcohol syndrome [149]. To date, five cohort or case–control studies have examined ASD risk through alcohol consumption more directly, indicating that mild to moderate maternal alcohol consumption poses no risk for autism [150–154]. Our review of the literature failed to identify any study examining the role of *paternal* alcohol use in the risk of autism.

### Nutrition

#### Interpregnancy interval

Maternal nutrition significantly influences the trajectory of fetal development and is particularly crucial during pregnancy [155] given it largely determines the nutrients available to support the growing fetus, placenta and maternal tissues. Deficient and malnourished diets can malign fetal programming and adversely impact developmental outcomes. Short intervals between pregnancies can tax a mother’s system with nutrients, particularly essential nutrients (9 amino acids, 2 fatty acids, 13 vitamins and 15 minerals), remaining low for months to up to a year after delivery [156]. The depletion of essential nutrients in the mother is associated with adverse health outcomes for offspring [157] including increased autism risk. In a review of seven studies (*N* = 1,140,210), short intervals between pregnancies bore an increased risk for any ASD (OR 1.90, 95% CI 1.16–3.09) with the association strongest for core autistic disorder (OR 2.62, 95% CI 1.53–4.50) [158].

#### Vitamin D

Multiple biological functions in the human body depend on vitamin D, including calcium homeostasis and metabolism, with mounting evidence that hypovitaminosis D is associated with a higher incidence of fetal miscarriage, preeclampsia, gestational diabetes, bacterial vaginosis, and impaired fetal and childhood growth and development [159]. Vitamin D receptors and enzymes are active in brain neurons and glial cells, pointing to a role of vitamin D in neurodevelopment in utero [160]. A recent systematic literature review examined seven areas of interest relevant to the understanding of the association between ASD and vitamin D including: latitude, season of conception and birth, maternal migration and ethnicity, the vitamin D status of mothers and ASD cases, and the role of vitamin D as an intervention in both the treatment and prevention of ASD [161]. This review concluded that there are indications that deficiencies in vitamin D during early development interacts with other risks, possibly contributing to the etiology of autism. There was also some evidence that vitamin D may have therapeutic benefits in reducing autism symptomatology among diagnosed cases. A later Swedish whole population register-based study found, that although rare, vitamin D deficiency was associated with offspring risk of ASD with, but not without, intellectual disability (ORs 2.51 and 1.28, 95% CI 1.22–5.16 and 0.68–2.42) [162].

#### Iron

Iron deficiency is common in pregnant women affecting up to half of all mothers [163], with maternal iron deficiency being causal in fetal iron deficiency [164]. Iron is crucial for neural function in general, and fetal development in particular, contributing to neurotransmitter synthesis, myelination, and immune function [165]. Findings examining a possible association between autism and iron deficiency are conflicting. In the CHARGE case–control study mothers with low iron intake had double the odds of having a child with ASD, especially in the presence of other autism risk factors (e.g., advanced age, diabetes, hypertension, obesity). However, this finding was not ratified in a Norwegian birth cohort [166, 167].

#### Zinc and copper

Deficiencies in maternal zinc during pregnancy can be harmful to fetal development having been identified as causal in neural tube defects and as possibly contributing to ASD risk [168, 169]. Low levels of zinc have been measured in the infant hair of individuals with ASD [170], and in mouse models zinc deficiency during development leads to alterations in social behavior [171]. Disruptions in fetal copper homeostasis during brain development might contribute to ASD risk, with both elevated and decreased copper levels linked with autism [172–174]. Employing a validated tooth matrix in a twin–cotwin design with monozygotic and dizygotic twins discordant for ASD, a study tested whether fetal and postnatal metal dysregulation increases ASD risk. Findings revealed significant divergences in metal uptake between ASD cases and their control twins during discrete developmental periods, and correlations between reduced zinc uptake and ASD severity and autistic traits [175]. These findings have been further corroborated in a follow-up study examining three independent teeth samples from the USA and UK, which identified the presence of alterations in fetal and postnatal zinc–copper rhythms in ASD in terms of cycling duration, regularity, and number of complex features [176].

### Vaccination

Despite strong evidence to the contrary, no hypothesized role for an environmental exposure in the etiology of autism has been pursued with as much sustained vehemence as that of the combined mumps, measles, and rubella (MMR) vaccination. Initially based on 12 cases of clinical gastroenterological symptoms, this now retraced study proposed a pathway from MMR vaccination to inflammatory bowel syndrome to ASD [177]. This study generated worldwide attention and belief, and is possibly the single most significant factor contributing to the harmful drop of vaccination rates and measles outbreaks across a range of countries, where measles was previously eradicated [178]. For more than a decade, a multitude of large-scale epidemiological studies have provided evidence refuting this notion [179, 180] including the role of vaccinations containing thiomersal in the ASD etiology [181]. Importantly, the initial paper has now proven to have been falsified in many aspects, including 3 of the 12 cases never being diagnosed with autism at all, 3 of 9 cases experiencing no regression, and all 12 cases reportedly typically developing prior to vaccination revealed to have preexisting developmental concerns [182]. Finally, it later emerged that the author of the initial study was paid to undermine the combined MMR vaccination by a lawyer attempting to raise a speculative class action lawsuit against drug companies manufacturing the triple vaccine [183], with the consequence that he was barred from practicing medicine in the UK.

### Toxic exposures

The modern world has generated a universe of some 80,000 environmental chemicals released from indoor (furniture, colors, building material, cosmetics) and outdoor sources (vehicles, industry, agriculture), with approximately 1000 of these demonstrating neurotoxicity and many others under or unstudied. Neurotoxins fall into the categories of air pollutants, heavy metals, persistent organic pollutants, pesticides, and non-persistent organic pollutants. Xenobiotic agents may act through diverse pathophysiological pathways in the immune, gut–brain and endocrine systems, interacting with genetic factors, thus altering the neurodevelopment of neural circuitry and synapses, cell migration and connectivity [184].

#### Air pollutants

There is a growing body of literature documenting the association between airborne pollutants and ASD, with epidemiological studies undertaken during the last decade recently summarized in several reviews [185, 186]. Adverse reactions linked to air pollution include neuroinflammation and oxidative stress [187–190], with a recent systematic review and meta-analysis identifying 23 studies examining its association with autism reporting ORs of 1.07 (95% CI 1.06–1.08) per 10-μg/m^3^ increase in PM10 exposure (*k* = 6 studies) and 2.32 (95% CI 2.15–2.51) per 10-μg/m^3^ increase in PM2.5 exposure (*k* = 3 studies) [191], concluding that modest evidence exists for the toxicity of air pollution during early development. These findings provide support for public health policies aiming to limit exposure to harmful airborne contaminants.

#### Heavy metals

Toxic metals occur both naturally and are produced by industrial processes, being present in ambient air, soil, water and plants, and medical products. Exposure to heavy metals can detrimentally impact many bodily functions, inducing neurological and behavioral impairment [191–195]. Several toxic metals bare a risk in the etiology of autism, in particular mercury and lead. A recent systematic review and meta-analysis examining the link between toxic metals and autism found 48 relevant case–control studies measuring levels of toxic metals (antimony, arsenic, cadmium, lead, manganese, mercury, nickel, silver, and thallium) in whole blood, plasma, serum, red cells, hair and urine) [196], with hair concentrations of antimony [standardized mean difference (SMD) = 0.24; 95% CI 0.03–0.45] and lead (SMD = 0.60; 95% CI 0.17–1.03) in ASD cases significantly higher than those of control subjects. ASD cases presented with higher levels of erythrocyte lead (SMD = 1.55, 95% CI 0.2–2.89) and mercury (SMD = 1.56, 95% CI 0.42–2.70), and higher blood lead levels (SMD = 0.43, 95% CI 0.02–0.85). Sensitivity analyses revealed that ASD cases in developed, but not in developing countries, had lower hair concentrations of cadmium (SMD = − 0.29, 95% CI − 0.46 to − 0.12). Similarly, analyses indicated that autistic individuals in low income, but not high-income countries, had increased lead concentrations in their hair (SMD = 1.58, 95% CI 0.80–2.36) and mercury (SMD = 0.77, 95% CI 0.31–1.23). For heavy metals in ambient air, positive and statistically significant effects have been found, although the effects are generally small and not consistent [191].

#### Pesticides

Herbicides, insecticides, insect repellents, animal repellents, antimicrobials, fungicides, disinfectants, and sanitizers are summarized under the label of pesticides. They are all agents discouraging pests and are explicitly designed to harm and kill organisms. Several of the active ingredients of these products target living organisms through their nervous systems, inhibiting acetylcholinesterase production in the brain, altering GABA neurotransmission [197, 198]. A review comprising of seven epidemiological studies conducted in 2014 noted that all studies documented an association across all classes of pesticides and ASD risk, with several associations reaching significance. These effects were the largest for exposures in weeks 1–7 of pregnancy, and postnatally in weeks 4–12 [185]. A more recent case–control study drawing on data from the CHARGE study [199] found that proximity to organophosphates during pregnancy was associated with a 60% increase in ASD risk. This risk was amplified for exposures during the third trimester (OR 2.0; 95% CI 1.1–3.6), and exposures to chlorpyrifos during the second trimester (OR 3.3; 95% CI 1.5–7.4). Pyrethroid insecticide exposure immediately prior to conception or during third trimester posed an increased risk for both ASD and developmental delay, with ORs ranging from 1.7 to 2.3.

#### Non-persistent organic pollutants

These toxins mainly include phthalates and bisphenol, used primarily in the production of plastics. While they do not persist in the human body, being at least partially cleared by bodily processes, their presence in the modern environment is ubiquitous, potentially posing a risk to the reproductive, respiratory, and endocrine systems, being possibly involved in carcinogenesis and adversely effecting neurodevelopment [200, 201]. The role of phthalates in ASD was recently reviewed and summarized across seven studies, inclusive of five human studies, three case–control in design and two cohort studies [202]. One cohort and two case–control studies reported an association between phthalate and autism. Concerning bisphenol A, the published literature, mostly characterized by smaller case–control studies is also conflicting, reporting associations ranging from none to rather substantial links with clinical autism and autistic traits [203–206]. A recent animal model study of maternal and paternal bisphenol exposure indicated behavioral effects in the area of anxiety, rather than social behaviors [207].

#### Persistent organic pollutants

Organic compounds resistant to environmental degradation accumulate in the environment and food chains with the potential to negatively influence human health, particularly through the consumption of animal fat and breast milk. The Stockholm Convention on Persistent Organic Pollutants was ratified in 2001, with the aim of banning these pollutants worldwide. A recent review summarized the evidence of the potential association between autism and autism relevant phenotypes and persistent organic pollutants for three major agents: dichlorodiphenyltrichloroethane (DDT), polychlorinated biphenyls (PCBs), and polybromated diphenyl ethers (PBDEs) [208]. Collectively, these agents have shown adverse endocrine, immune, and neurodevelopmental effects in humans [209]. Two studies have investigated the influence of the pesticide DDT on neurodevelopment in humans and rats, demonstrating a negative impact on cognitive skills (IQ, memory) and gene expression in the hypothalamus [210, 211]. Studies on PCBs, previously used in coolant fluids in electrical apparatus, have focused on cognitive skills, demonstrating negative effects on various intellectual, motor and verbal outcomes of relevance to autism [212, 213]. A recent larger case–control study also found organochlorine compounds during pregnancy were associated with ASD [214]. While PBDEs, historically used as fire retardants in furniture and other products negatively impact on neurodevelopment [215], the CHARGE study reported no differences in plasma PBDE levels in autism and typical control cases [216].

### Psychosocial factors

Owing to the long-lasting false hypothesis of a psychogenic causation of autism [217], any possible contributions of the psychosocial environment to autism etiology have been largely avoided by research. However, conceptualizations of mental disorders and maladaptation must not stop at the individual, but be understood at a societal level, considering the potential mismatch between an individual’s skills and needs and societal expectations and demands. It is well known and accepted that the psychosocial environment, independent of etiological considerations, plays a role in modifying the severity, quality of life and functional outcomes or level of impairment associated with ASD. Access to early identification and intervention, supportive and understanding environments maximizing adaptation to an autistic individual’s needs (“inclusion”), and appropriate education and employment are critical in determining functional abilities and disabilities [3, 218]. Psychosocial factors may also have a role beyond purely modifying outcomes. For instance, it is now established in human and animal models that intense maternal stress during pregnancy may have long-term biological and behavioral effects on the child [219–221]. In addition, extreme deprivation in infancy such as that experienced in institutions with impoverished levels of care, stimulation, and attention may have adverse effects on developmental psychopathology and physical development [222].

#### Maternal migration

Neurodevelopmental biological correlates associated with prenatal stress in offspring include cognitive function, cerebral processing, and functional and structural brain connectivity involving amygdalae and (pre)frontal cortex, changes in hypothalamo-pituitary–adrenal axis and the autonomous nervous system [223]. In autism, the major life event of maternal immigration has been the most studied cause of maternal stress, and possibly associated with immune activation (see above). An alternative explanation, not linked to stress, is reduced vitamin D levels in dark skinned migrants moving to the northern hemisphere [224]. Results examining the association between maternal immigration and autism are mixed. A 2015 review including ten studies found a positive association with immigration in three studies, no connection in five, and a reverse association in two studies [225]. Six of the ten studies found that giving birth postmaternal migration increased ASD risk. A large registry study from Sweden, not included in the review, reported that third trimester prenatal stress increased ASD risk (OR 1.58, 95% CI 1.15–2.17) [226]. Another later Finnish hospital discharge diagnosis study on the matter, focusing on Asperger syndrome only, found a reduced risk in immigrant families, including those from Sub-Saharan Africa [227].

#### Natural disasters

The association of exposure to prenatal maternal stress (PNMS) and ASD risk or ASD-related cognitive features has been studied in the context of natural disaster cohorts that mimic the random allocation of experimental designs. The Project Ice Storm, the QF2011 Queensland Flood Study, and a study on the association between the prevalence of ASD and tropical storms in Louisiana are examples of this research approach [221, 228, 229]. In the Project Ice Storm [221], mothers’ objective stress, and subjective distress during the early stages of pregnancy explained between 23 and 42.7% of the variance in autistic symptoms in 6-year-olds. Exposure to tropical storms in Louisiana [228] from 1980 to 1995 was employed as a model, examining if risk for ASD increases in a dose–response pattern parallel with the severity of PNMS (as inferred from storm severity), and sensitivity of gestational periods to ASD risk. While a dose–response relationship for ASD emerged across cohorts, in contrast to the findings of Project Ice Storm, this was particularly strong for exposures occurring during the middle and end stages of gestation. The QF2011, Queensland Flood Study [229] examined the association between PNMS and theory of mind challenges. Higher subjective stress, but not objective hardship predicted poorer theory of mind skills in 130 children at 30 months of age.

#### Institutional deprivation

Children adopted from globally deficient orphanages may initially show a variety of atypical behaviors, including stereotyped self-stimulation, inability to form deep or genuine attachments, indiscriminate friendliness, and difficulty establishing appropriate peer relationships. Severe early deprivation may also be a major contributor to delayed development and longer-term extreme behaviors, with such experiences possibly particularly impactful between 6 and 18 months of life [222]. The Romanian adoptee study compared 144 children initially raised in Romanian institutions, but then adopted by UK families, and later followed up at ages 4, 6, and 11 years with a non-institutionalized sample of 52 domestic adoptees. Sixteen of the Romanian children were found to have “quasi-autism” with additional children presenting with autistic features, while none of the domestic adoptees presented with any signs of autism. However, by age 11, a quarter of the children had “lost” their autistic-like behaviors, with the remaining children demonstrating both similarities and differences to classic ASD [230]. Importantly, despite their early extreme deprivation, only a minority of cases developed quasi-autism with the majority recovering from their early experiences. These findings are of limited relevance to understanding the etiology of autism outside of institutional settings given that symptoms resulted from exposure to extreme psychosocial deprivation.

### Protective factors

While the overwhelming majority of research has examined environmental risk in autism, there is an emerging body of research examining the role of potentially protective factors, largely from the field of nutrition and food supplementation, with several underpinned by findings from the risk factor literature. Studies indicate prenatal vitamin supplementation close to delivery might reduce the risk of autism in offspring, with folate (vitamin, B9, folic acid, folacin) receiving considerable attention [231–233]. Folate is essential in the production and maintenance of cells, to DNA and RNA synthesis and methylation, in preventing changes to DNA and various other cellular processes, and centrally involved in cancer prevention [234]. There is wide evidence that pre and periconceptional folate supplementation supports neural and neurobehavioral development, bolstering social, cognitive and verbal functioning [235–237]. It has been hypothesized that in autism, folate may act as a methyl donor, supporting remethylation during early embryogenesis [232, 233]. While folate is generally considered not to protect against ASD, it may buffer additional risks, such as in mothers or infants who are carriers of gene variants impacting the efficiency of folate-dependent one-carbon metabolism or fetuses with neural tube alterations.

Fatty acids including the omega-3 group are assumed to play a key role in neurodevelopment during early childhood as well as in regulating cognitive functioning across the life span [238], with supplementation studies revealing their benefits to neural efficiency [239]. In autism, the few studies examining the effect of maternal fatty acid supplementation or intake on autism and autistic trait outcomes have revealed inconsistent results. This research is equally counterbalanced, with two studies each both identifying and failing to identify an association between maternal omega 3/omega 6 intake or status and autism related outcomes [240–242]. The many connections between brain functioning and development, and gastrointestinal functioning, have been increasingly highlighted in the field of psychiatry, and specifically in ASD [243, 244]. The role of the gastrointestinal tract, the largest immune organ in the human body, and the role of several aspects of immunity and inflammation potentially relevant to the etiology of ASD have been discussed earlier in this review. There is evidence that some probiotic bacteria migrate from the mother to the child [245], with probiotic supplementation during pregnancy a promising, but as yet unexplored field of future investigation in autism protective factors [246].

## Discussion

Research examining the etiology of autism across the last 25 years has been dominated by a focus on genetic factors; however, there is increasing awareness of the potential significance of environmental influences in the etiology of ASD. The results of recent twin and family studies point toward a greater role for environmental contributions [29, 30], with pessimism toward such approaches decreasing, some of which was historically fueled by fake research in the area including unproven claims of vaccinations being causal of ASD [177]. In addition, given the global increase in diagnoses rates of ASD and the increasing availability of funding for research, researchers from all fields of environmental science are more and more engaging in autism research.

In this review, an up-to-date overview of the potential environmental contributions to autism development and presumed pathophysiological mechanisms is provided for parental age, several aspects of the immediate fetal environment, obstetric complications, medication during pregnancy, smoking and alcohol use, nutrition, diverse toxic exposures, as well as protective nutritional factors and the role psychosocial factors. Evidence, both positive and negative, is mounting in relation to the role these risks play in the etiology of ASD. Several areas are now underpinned by relatively large bodies of evidence such as parental age and SSRI medication [58, 130–132], while others lines of inquiry have generated relatively little specific evidence such as smoking and alcohol use [145, 146, 150–154].

In the presence a plethora of existing agents, evidence regarding the impact of environmental toxins on human health and development in general, and autism in particular, is lacking. Although the generalizability of animal studies to humans remains relatively unknown, animal models have yielded many intriguing insights into the effects and mechanisms of inflammation and immune activation, as well as the role of toxic agents, in inducing autism-like behaviors in rodent and other species [103, 104, 112–115, 207]. Overall, understanding the role of environmental exposures in the etiology of ASD is a broad and complex field, still largely in its infancy with many current limitations, but also with many future opportunities.

### Limitations

The etiology of ASD is heterogeneous, as are its phenotypes. It is both a clinically relevant phenomenon, and a quantitative trait in the general population, with broader subclinical phenotypes frequently present in relatives [247, 248]. In clinical cases, other neurodevelopmental conditions, psychiatric disorders and somatic disorders are often co-occurring complications [13–15]. Finally, diagnoses rates have risen dramatically in the last 20 years, with a parallel widening of the diagnostic concept undoubtedly one of the driving factors. The etiology of ASD is complex, with causal factors unknown in many cases. Collectively, these factors make research aimed at improving our understanding of the etiology of pure autism challenging. Today, up to 15% of autism variants can be linked to genetic determinants, with future projections that as much as to 50% of genetic etiologies are discoverable using sequencing approaches [26]. Even if this is realized, it leaves considerable space for speculation as to the etiological role of environmental contributions. Exposure to many factors occurs at a population level, such as in the case of air pollutants, with their role in autism etiology far from fully understood. While still largely unexplored, some effects may result from interactions between environmental factors and genes acting to increase ASD risk, with several intriguing examples emerging such as between MET rs1858830 CC genotype on the one hand, and early life stress and air pollutant exposure on the other [249, 250]. A related issue is the importance of epigenetic modulation, such as alterations in DNA methylation through which PCBs, lead, and bisphenol confer a risk for ASD [251].

Reported autism–environment associations may not be causal, for example there is doubt as to the causal role of obstetric complications, which are perhaps more likely to be epiphenomena of primary genetic risks [122]. In addition, parental age might be confounded by parents with autism traits having children later in life, or choosing partners with high autism traits [252, 253]. Maternal SSRI risks might be confounded with maternal depression diagnosis and broader autism phenotypes [254]. These are only a few examples of the multitude of possible confounders of environmental risk factors. Many have received little attention in research, such as the cultural bias apparent in the association of migration and autism risk, with one study reporting a reduced risk of Asperger syndrome among immigrant families in Finland. Such findings might be explained by cultural and familial factors with clinical experience suggesting that milder variants of ASD might not be perceived as equally atypical or impairing by Finnish and immigrant families (with immigrant families having a higher threshold for perceiving deviance), leading to referral and diagnostic bias. It might also be possible that neurodevelopmental disorders are more stigmatized among immigrants, leading to a tendency to avoid clinical assessment. Another challenge, remaining largely unaddressed, is the additive and interactive effects across environmental factors. For instance, a recent study showed that associations between pesticide exposures and ASD were modulated by folate intake during the first month of pregnancy [255] with evidence of a cumulative risk for environmental effects in autism [32].

Published research in this field has many shortcomings in relation to design, with the majority of studies being retrospective, cross-sectional, case–control, and cohort approaches, with very few employing experimental or prospective designs with a priori statement of hypotheses restricting conclusions in relation to causality. Further, the majority of case–control studies have been small with imprecise measures of exposure. For example, research examining air pollution exposure employing indirect measures of exposure (such as distance to a freeway where emissions were measured) have reported associations to autism [256], while studies measuring emission levels close to the individuals’ homes have not [257]. Further, this line of research should consider the role of both cultural factors and residential area in confounding the relationship between pollution exposure and autism, a notion consistent with positive associations originating largely from the USA and negative results emerging from Europe, with socioeconomic status likely to play a role in both. As in other fields of autism research, there are high levels of variability in outcome assessments, ranging from register-based to clinical gold standard, with both categorical and dimensional scales, limiting comparability. Given the evolving nature of autism as a concept and changes in community awareness and understanding, it might be difficult to compare older and new studies in terms of the autism measured.

### Opportunities

Viewed optimistically, the various limitations outlined above provide many opportunities in directing and improving future research. Key to understanding the role of environmental factors in the etiology of autism is mapping the critical time points of vulnerability in pregnancy and early development. Alterations to neural migration, laminar disorganization, neuron maturation and neurite outgrowth, synaptogenesis and reduced neural network functioning likely play a crucial role in autism development [258], with varying levels of susceptibility to adverse environmental influences during various stages of pregnancy. These critical time points are still largely unmapped for most agents in relation to their risk of ASD, with points likely to vary across environmental hazards. While an increasing number of studies have attempted to address this issue, for instance in regards to the role air pollution plays in ASD risk [259], findings remain insufficiently robust to support firm conclusions. A likely fruitful line of research would be for basic human research and neighboring fields to focus explicitly on the risk various environmental exposures pose for ASD across the stages of pregnancy [260].

Clearly, more cross-discipline research is needed to understand autism etiologies. Multi-hit models of ASD included genetic and environmental factors and their interaction. While they are theoretically well-accepted, the empirical evidence of their causality in ASD remains weak. The literature contains several attempts to design such risk models, for example in examining the association between genetic disposition and maternal antidepressant use [261]. Despite the evidential methodological challenges, the utility of these multiple hit models in understanding ASD etiology should be further explored.

While research to date has largely focused on examining the environmental risk factors of ASD, examining environmental protective factors might be equally, if not more valuable. Despite wide speculation, very few protective factors have been systematically studied in ASD, and evidence is emerging as to the potentially protective role folate and other nutritional factors might play in buffering ASD risk. This line of research presents many opportunities including the identification of mechanisms of prevention and potential interventions. For instance, the finding in mice models that risk for obese mothers to have offspring with behavioral problems linked with autism is ameliorated by dietary intervention during pregnancy and lactation might be translatable to humans [262, 263].

A promising and still largely ignored line of environmental research relates to trends in the prevalence of autism. Specifically, data from Swedish regional registries [18] show that the increase in ASD rates observed in high-income countries in recent years is almost exclusively accounted for by ASD in the normative intellectual range, while ASD linked with intellectual disability is decreasing. Other surveillance systems, such as the Center of Disease Control Autism and Developmental Disabilities Monitoring (ADDM) Network in the USA (cdc.gov/ncbddd/autism/addm.html), have also identified this trend. Further, ASD risk associated with environmental exposures has been stable for air pollutants and pesticides, with lifestyle risk factors such as smoking and alcohol consumption and other potential hazards related to nutrition and medication in decline [264]. The potential association between the downward trajectory in the number of ASD cases with associated intellectual disability and the curtailing of several environmental risk factors is worth investigating. While ASD presents across all levels of intellectual functioning, the distinction between ASD with and without challenges is commonly made on the basis of intellect, making this an important line of research to pursue. Stratifying ASD on the basis of factors other than solely intellect, generating more homogenous groups, would still likely support more conclusive findings in relation to the etiology of autism. However, despite effort to subtype ASD and look at environmental and genetic risk factors within subtypes including sex, comorbidities, verbal abilities, neurocognitive and biological endophenotypes, these attempts have thus far not been very fruitful [265]. Nevertheless, there are still multiple options for stratification to be explored. Several major collaborative efforts in ASD, such as the EU-AIMS (eu-aims.eu) specifically aim to understand biomarkers potentially relevant to stratification [266–268]. Other ongoing longitudinal large-scale projects such as the Norwegian Mother and Child Cohort Study (fhi.no/moba-en) [269],the Swedish Lifegene Study (lifegene.se) [270] or the National Institutes of Health Environmental influences on Child Health Outcomes Program (nih.gov/echo) offer opportunities to study the environmental risk factors in autism and other conditions in detail.

Although ASD is no longer considered a rare condition, autism research is challenged by studies employing small sample sizes. The evolving body of autism research shows that autism is not a tightly bounded clinical entity, but that traits from low to extreme exist more broadly in the general population. Viewing autistic phenotypes as continuous, rather than categorical, provides an opportunity to underpin studies with larger samples, increasing their sensitivity to small and medium effects. Finally, twin studies provide a unique opportunity to examine both the genetic and environmental contributions to ASD etiology. In particular, contrasting the phenotypes of discordant and monozygotic twins enables control of genetic factors, providing a powerful strategy to identify disease-associated environmental factors, independent of underlying genomic sequence variation [271]. The Roots of Autism and ADHD Twin Study Sweden (RATSS) [272], a sub-study of the population-based Child and Adolescent Twin Study Sweden (CATSS) [273], is the largest collection of deeply clinically phenotyped autism twins. It primarily applies twin–cotwin analyses to identify environmental risks contributions to autism phenotypes on the behavioral and neurobiological level controlling for genetic and familial factors. It has generated several novel findings and hypotheses related to the role of non-shared environment in ASD, such as the potential significance of altered zinc-copper cycles and dysregulation of other essential and toxic metals during critical pre- and postnatal developmental windows [32, 175, 176, 274, 275].

In conclusion, this review provides a broad and updated review of the potential environmental risks in the etiology of autism, discussing the limitations of current research and identifying likely fruitful pathways for future research. The majority of current research is preclinical in design, limiting its ability to inform prevention and intervention strategies in the real world. The ultimate goal of all medical research must be to make discoveries that improve people’s lives. Hopefully, future research aimed at understanding the role of environmental factors in the etiology of ASD will reach this stage. The current review shows that designing population-level studies, informed by findings from basic research, is likely to work toward achieving this goal.
